# Rescue therapy for recurrent cholangitis secondary to main duct intraductal papillary mucinous neoplasm with pancreatobiliary fistula using an esophageal fully covered metal stent

**DOI:** 10.1055/a-2619-6966

**Published:** 2025-07-15

**Authors:** Anne Kimberly Lim-Fernandez, Samuel Jun Ming Lim, Christopher Jen Lock Khor, Damien Meng Yew Tan

**Affiliations:** 1 37581 Department of Gastroenterology and Hepatology, Singapore General Hospital, Singapore, Singapore; 2 Department of Internal Medicine, Metro Davao Medical and Research Center, Philippines; 3 121579 Duke-NUS Medical School, Singapore, Singapore

Main duct intraductal papillary mucinous neoplasms (MD-IPMNs) of the pancreas may be complicated by fistula formation 1 and biliary obstruction from excessive mucin 2 . Fistulation occurs in 6.6% of patients, involving organs like the common bile duct (CBD) 3 .

We present a 78-year-old man diagnosed with MD-IPMN who declined surgery and defaulted follow-up. Four years later, he presented with cholangitis. A computed tomography (CT) scan showed a 61 × 29-mm pancreatic mass with liver metastases and a 25-mm dilated CBD with fistulation between the main pancreatic duct and the mid-CBD ( Fig. 1 ). The patient underwent endoscopic retrograde cholangiopancreatography (ERCP) and insertion of a 10 × 60-mm biliary fully covered self-expanding metal stent (FCSEMS) (WallFlex; Boston Scientific, Marlborough, Massachusetts, USA) and a 7-Fr × 12-cm double-pigtail stent (Zimmon; Cook Medical, Bloomington, Indiana, USA) within the FCSEMS.

**Fig. 1:**
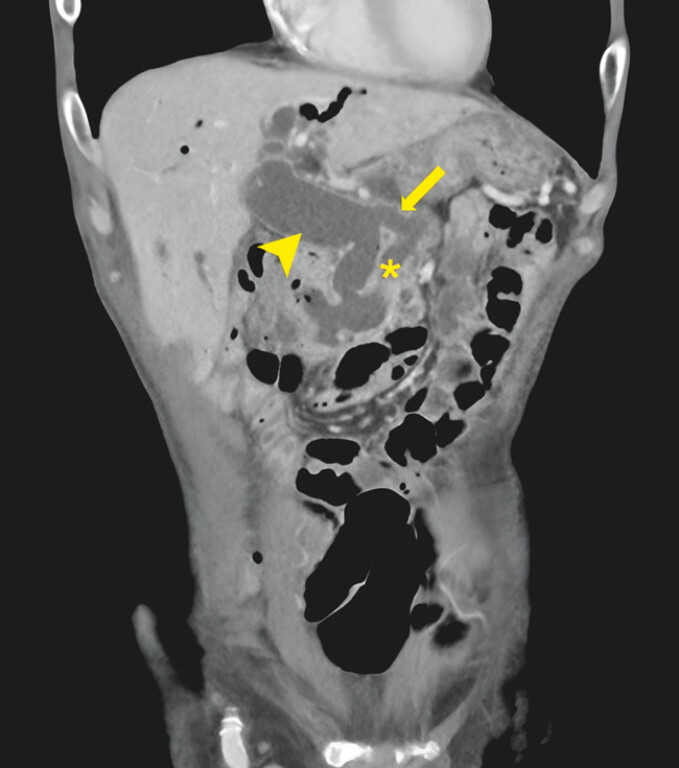
Main duct intraductal papillary mucinous neoplasm with pancreatobiliary fistula. Dilated common bile duct (arrowhead), dilated main pancreatic duct (asterisk), pancreatobiliary fistula (arrow).

He had initial clinical improvement but developed recurrent cholangitis two weeks later. A repeat CT scan showed proximal migration of the biliary FCSEMS ( Fig. 2 ). The use of an 18 × 97-mm through-the-scope esophageal FCSEMS (Agile; Boston Scientific) for repeat biliary stenting was considered because of its larger diameter. Informed consent was obtained from the patient after the off-label use with procedural risks, including perforation, was explained. Repeat ERCP was performed to remove the migrated biliary FCSEMS and double-pigtail stent, followed by balloon sweeps to remove excessive mucin within the 30-mm dilated CBD ( Video 1 ). The esophageal FCSEMS was successfully deployed in the CBD to close the pancreatobiliary fistula. A 7-Fr × 12-cm double-pigtail stent was then placed within the FCSEMS to prevent stent migration ( Fig. 3 ). The procedure lasted 45 minutes with no post-procedure complications. The patient recovered well and opted for the best supportive care.

**Fig. 2:**
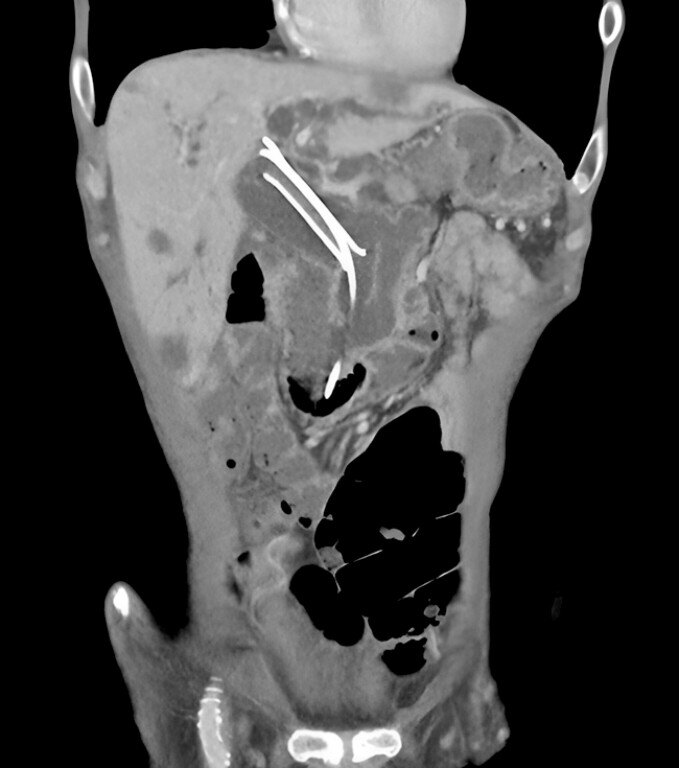
Proximal migration of biliary fully covered self-expanding metal stent (FCSEMS).

Removal of migrated biliary fully covered self-expanding metal stent, extraction of large amount of mucin from a dilated common bile duct, followed by insertion of a through-the-scope esophageal fully covered self-expanding metal stent in the common bile duct.Video 1

**Fig. 3:**
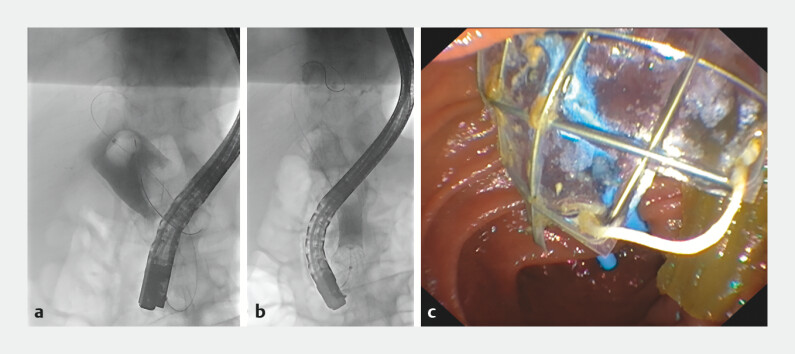
**a** Dilated common bile duct with a 20-mm extraction balloon and excessive mucin within. **b** Occlusion cholangiogram after deployment of esophageal FCSEMS. **c** Endoscopic view of esophageal FCSEMS and biliary double-pigtail stent after placement.

Off-label use of esophageal FCSEMS may be considered for biliary stenting of a severely dilated CBD as smaller stents may be prone to migration.

Endoscopy_UCTN_Code_TTT_1AS_2AJ

## Notes

Endoscopy E-Videos https://eref.thieme.de/e-videos
*E-Videos* is an open access online section of the journal *Endoscopy* , reporting on interesting cases and new techniques in gastroenterological endoscopy. All papers include a high-quality video and are published with a Creative Commons CC-BY license. Endoscopy E-Videos qualify for HINARI discounts and waivers and eligibility is automatically checked during the submission process. We grant 100% waivers to articles whose corresponding authors are based in Group A countries and 50% waivers to those who are based in Group B countries as classified by Research4Life (see: https://www.research4life.org/access/eligibility/ ). This section has its own submission website at https://mc.manuscriptcentral.com/e-videos .
